# The effects of endurance training on muscle oxygen desaturation during incremental exercise tests: a systematic review and meta-analysis

**DOI:** 10.3389/fspor.2024.1406987

**Published:** 2024-10-24

**Authors:** Assaf Yogev, Jem I. Arnold, Hannah Nelson, Michael A. Rosenblat, David C. Clarke, Jordan A. Guenette, Ben C. Sporer, Michael S. Koehle

**Affiliations:** ^1^ Environmental Physiology Laboratory, The University of British Columbia, School of Kinesiology, Vancouver, BC, Canada; ^2^ Department of Biomedical Physiology and Kinesiology and Sports Analytics Group, Simon Fraser University, Burnaby, BC, Canada; ^3^ Department of Physical Therapy, The University of British Columbia, Vancouver, BC, Canada; ^4^ Centre for Heart Lung Innovation, Providence Research, The University of British Columbia and St. Paul’s Hospital, Vancouver, BC, Canada; ^5^ Vancouver Whitecaps FC, Vancouver, BC, Canada; ^6^ Division of Sport & Exercise Medicine, The University of British Columbia, Vancouver, BC, Canada

**Keywords:** NIRS (near infrared reflectance spectroscopy), muscle oxygenation, endurance training, incremental exercise test, systematic review

## Abstract

**Objective:**

Minimum muscle oxygen saturation (SmO_2_min) measured via near-infrared spectroscopy (NIRS) is a common measure during incremental exercise testing (IET). Our objective was to determine the effects of pre-to-post endurance training on SmO_2_min (ΔSmO_2_min) during an IET, using a meta-analysis.

**Data sources:**

MEDLINE, EMBASE, and SPORTDiscus.

**Study selection:**

Studies including healthy individuals had to meet the following criteria: (1) endurance training intervention; (2) peripheral muscle NIRS; (3) incremental exercise test pre/post training; (4) SmO_2_ or analogous saturation parameter measured.

**Analysis:**

A PEDro scale was used for risk of bias analysis. A random effect meta-analysis model was used to synthesize the effect of training on ΔSmO_2_min in individual studies. Statistical heterogeneity was quantified using *I^2^* statistic. A meta-regression was used to estimate the effect of training on the relationship between peak cycling power output (Wpeak), peak pulmonary oxygen uptake (V˙O_2_peak), and ΔSmO_2_min. A mixed-effect model was used to estimate categorical variables.

**Results:**

Five studies met the inclusion criteria. No difference in SmO_2_min was detected following training pre- and post-intervention IETs. A trend for an effect of training on the relationship between Wpeak and ΔSmO_2_min was observed (*p *= 0.06).

**Conclusion:**

This meta-analysis showed no effects of endurance training on SmO_2_min during an IET. Our results showed a trend for an effect of training on the relationship between Wpeak and ΔSmO_2_min, with no effect for V˙O_2_peak and ΔSmO_2_min. It is possible that SmO_2_min is not affected by endurance training, and may be used as a physiological marker for improvements in submaximal performance rather than at peak.

## Introduction

In healthy individuals, exercising at severe and extreme intensities can cause muscle oxygen demand to surpass convective oxygen delivery, thus causing a mismatch between muscle oxygen demand and supply (1). Following endurance training, physiological adaptations occur both for systemic oxygen delivery and muscle oxygen extraction and utilization, reducing the oxygen mismatch and improving overall athletic performance (2–4). Such improvements from pre- to post-training are often assessed by measuring the intensity-dependent response of various physiological and performance measures during incremental exercise tests (IET).

During an IET, a gold standard measurement of such physiological responses is systemic oxygen consumption (V˙O_2_), which demonstrates an intensity-dependent increase until peak V˙O_2_ is attained (V˙O_2_peak) (5). Conversely, another physiological measure is muscle oxygen saturation (SmO_2_), measured at the primary locomotor muscle using near-infrared spectroscopy (NIRS), which typically follows a non-linear but generally continuous desaturation (i.e., decreasing oxygen saturation) response until maximal task tolerance where a minimum SmO_2_ (SmO_2_min) is observed (6–8). Near-infrared spectroscopy has been used alongside peak cycling power output (Wpeak) and V˙O_2_peak during IET to describe and investigate local/peripheral metabolic responses alongside systemic physiological responses and performance outcomes (7–10). Maximal task tolerance during an IET is associated with the attainment of V˙O_2_peak and Wpeak (11, 12). Both measures have been shown to increase with endurance training (13). Muscle oxygen saturation following endurance training has been investigated in relation to these markers, showing a greater desaturation to a lower SmO_2_min at maximal task tolerance, coincident with a rise in V˙O_2_peak and performance (14). The integration of NIRS during an IET has increased in popularity within the last 20 years. As such, it will be valuable to systematically review the existing literature and quantify the effects of endurance training interventions on SmO_2_ during an IET.

The primary signals measured via NIRS are the relative concentration of oxygenated and deoxygenated hemoglobin and myoglobin (O_2_HbMb and HHbMb, respectively), and their sum (tHbMb) (15). The relative portion of O_2_HbMb from tHbMb is a measure of muscle oxygen saturation (SmO_2_). SmO_2_ may be estimated from different methods depending on the specific NIRS technology (15, 16). The saturation parameter may alternatively be called muscle or tissue oxygen saturation, saturation index, or oxygenation index, which reflect slightly different models of how the NIRS signal interacts with the tissue volume under illumination (15). However, the response profiles during an incremental exercise test (IET) are broadly comparable, so for clarity, the term SmO_2_ will be used throughout this manuscript.

For athletes, coaches, and sports scientists, the primary benefit of measuring SmO_2_ response during exercise is the ability to observe muscle metabolic responses within locomotor muscles. This aids in tactical decision-making during exercise, quantifying the relationship between exercise demands and internal muscle perturbation, and providing more information about muscular adaptations to training. Several studies reported breakpoints in the NIRS signal during an IET, relating them to other physiological thresholds such as lactate and ventilatory thresholds (9, 17, 18). Others have looked at SmO_2_min, V˙O_2_peak, and Wpeak during IETs to identify the effects of endurance training interventions on muscle metabolic responses over time (19–23). For practitioners, these reports highlight the benefit of NIRS as a non-invasive measure in estimating the effects of training on SmO_2_, especially with the evolution of commercially available wearable NIRS technology (24, 25). For our purposes, reviewing and quantifying the existing body of knowledge concerning the effects of endurance training on SmO_2_min during IETs, will address an important question: what change, if any, will SmO_2_min present following an endurance training intervention during an IET.

As such, in the context of this review, endurance training was defined as an aerobic exercise regimen that lasted for at least 2 weeks, with a minimum of two sessions per week that were composed of continuous modes of exercise (26). Strength exercise regimes were not included unless completed in addition to endurance training as previously defined. Endurance training interventions often include either continuous training sessions or interval training sessions, or a combination of the two (27, 28).

### Purpose

From previous studies evaluating changes across different SmO_2_ signals following endurance training interventions, we hypothesized that endurance training would induce greater desaturation to a lower SmO_2_min at maximal task tolerance during an IET. As for the effects of training on the change in SmO_2_min relative to Wpeak and V˙O_2_peak, we hypothesized that both Wpeak and V˙O_2_peak would increase with training and that a lower SmO_2_min would be correlated with higher post-training Wpeak and V˙O_2_peak. The aim of this meta-analysis was to provide an estimate of the effects of endurance training on SmO_2_min during an IET in healthy participants.

## Methods

The design of the review was based on Preferred Reporting Items for Systematic Reviews and Meta-Analysis (PRISMA), following the 2020 guidelines for new systematic reviews and the Cochrane Handbook for Systematic Reviews of Interventions version 6.3 (29) which included searches of databases, registers, and other sources (30).

### Identification of studies search strategy

The online databases MEDLINE (OVID), EMBASE (OVID), and SPORTDiscus (EBSCOHost) were searched for published, full-text articles in English up to and including July 2024. The keywords used to identify relevant studies can be found in Supplementary 1. Further records were identified by manual citation searching through additional online searches. The inclusion criteria were as follows: (1) were in English; (2) endurance training intervention (31); peripheral muscle NIRS (32); IET performed pre/post training (33); SmO_2_ or analogous saturation parameter measured. Exclusion criteria were as follows: (1) No clinical populations were included in the analysis, but clinical studies that had healthy controls with an endurance training intervention were included, with only the control data included in the extraction; (2). Studies were excluded if they had with no measure of muscle oxygen saturation or provided no data that allowed for calculation of saturation.

If reports included partial data, respective authors were contacted to provide additional data by the lead author (AY). If no response was received, a follow-up email was sent, and studies were excluded if no data were provided after 30 days. In the case where necessary data were only presented in a graphical format, PlotDigitizer (https://plotdigitizer.com, 3rd version, August 2022) was used to extract relevant values.

All records screened independently using Covidence systematic review software, Veritas Health Innovation, Melbourne, Australia, for eligibility by two authors (AY, JA, or HN), any disagreements were discussed and evaluated by the group (AY, JA, HN, and MK). Similarly, reports assessed for eligibility were independently reviewed by two authors (AY, and HN) and reason for exclusion was decided collaboratively (AY, and HN).

### Data extraction

**Figure 1:**
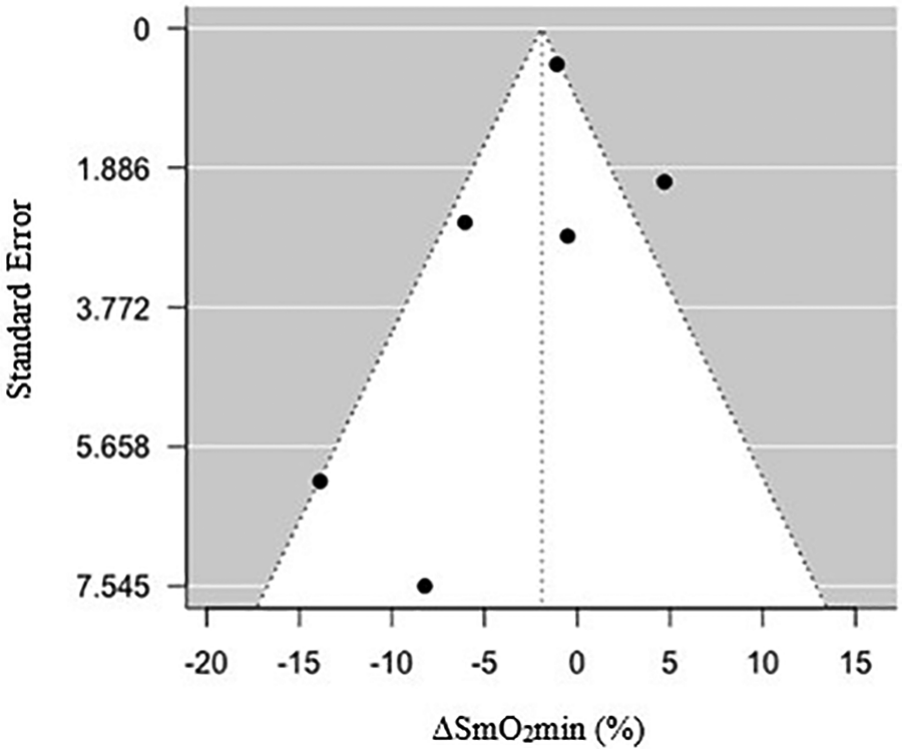
Funnel plot of standard error relative to a percentage change in minimum muscle oxygen saturation (ΔSmO_2_min) (%) following endurance training.

Data were initially extracted for a systematic review from reports assessed for eligibility by three authors (AY, HN, and JA) (Figure 1). However, for the five papers included in the quantitative synthesis, further data extraction and analysis was conducted by AY, HN, and MR.

The extracted data from each included article were: participant characteristics (sample size, sex, age, body mass, and BMI), intervention (exercise type, exercise mode, duration of training, frequency of training, and number of sessions), NIRS (device, baseline IET SmO_2_ pre-training, IET maximal task tolerance SmO_2_ pre-training, baseline IET SmO_2_ post-training, IET maximal task tolerance SmO_2_ post-training), Wpeak (IET Wpeak pre-training, IET Wpeak post-training), V˙O_2_peak (IET V˙O_2_peak pre-training, IET V˙O_2_peak post-training). Baseline IET SmO_2_ (%) showed no significant differences pre- to post-training in any of the included studies. Since there were no baseline differences, we normalized baseline SmO_2_ to 100% and differences from baseline during exercise were reflected as a decrease from 100% to the SmO_2_min (i.e., the lower the SmO_2_min value the more oxygen desaturated). To estimate the magnitude and direction of change in the standardized SmO_2_min between pre-training and post-training, the standardized SmO_2_min values were subtracted from each other to yield the change (ΔSmO_2_min). For Wpeak and V˙O_2_peak, if a *p*-value was not provided for change following training, it was estimated using the means and standard deviation (SD) from pre-training to post-training. When IET was longer than 12 min, a correction factor was used to standardize Wpeak (34).

### Risk of bias analysis

Risk of bias for individual studies was evaluated using the PEDro scale and the completed scale can be found in Supplementary 1. Two reviewers independently assessed studies for bias (AY and HN), and any disagreements were resolved by the reviewers and a third author (MK).

### Statistical analysis

Data analysis and statistical analysis were carried out in R (v4.1.2, R Foundation for Statistical Computing, Vienna, Austria). Group data were reported as means and SDs. The effect measure of the continuous primary outcome variable (ΔSmO_2_min) was presented as the standardized mean difference (SMD) and its 95% confidence intervals for pooled data, following standardization of the data as described above. When SD was not provided, standard error of the mean (SE) was converted using the following formula: $\mathrm{SD}=\mathrm{SE}\cdot \sqrt{n}$. When SE was not reported, the *p*-value was used to estimate the SD using the following formula: $\mathrm{SD}=\sqrt{n}\cdot \left(\frac{{\underline{x}}_{1}-{\underline{x}}_{2}}{t}\right)$. To provide a conservative estimate of the SD, a *p*-value expressed as an inequality (i.e., “<”) was changed to an equality (i.e., “=”) (27). A forest plot was produced to visually display the quantitative results; a random effect meta-analysis model (DerSimonian-Laird random effects method) was used to synthesize the individual studies (31). Statistical heterogeneity was quantified using the *I*^2^ statistic. *I*^2^ values of 25%, 50%, and 75% were considered as low, moderate, and high degrees of statistical heterogeneity (35). To determine the relationship between the effect size and the sample size, a funnel plot was used. An Egger's Test was used to account for any small sample size bias (36).

We conducted 5 subgroup analyses to estimate the effect of endurance training on ΔSmO_2_min in relation to the following modifiers: continuous variables (Wpeak and V˙O_2_peak) and categorical variables (sex, training type, and exercise mode). The expected effect of endurance training on the continuous variables is an increase, which was explored using meta-regressions and visually displayed as bubble plots. For these variables, the data were converted to a percentage change from pre-training to post-training. Categorical variables of the retrieved studies were evaluated based on sex (female or male), training type (continuous and interval exercise), and mode (leg cycling, combined arm and leg cycling, and unsupervised training). They were analyzed using a mixed-effects model to estimate an effect of endurance training on the relationship between ΔSmO_2_min and each of the categorial variables separately.

## Results

### Descriptive data

A combined total of 869 records were identified in the search prior to the removal of 366 duplicates by an automatic review application (Covidence systematic review software, Veritas Health Innovation, Melbourne, Australia) (Figure 1). After title and abstract screening, 25 reports were retrieved for full-text text review. One could not be retrieved for full text (37). Nineteen papers were excluded at the full text review stage. Eleven were excluded due to exercise protocol used (32, 38–47). Eight were excluded due to ineligible reported outcome measures (33, 48–54). Identification of studies via other methods yielded six studies. Five were ineligible due to exercise protocol used (55–59). Five studies were included in the final quantitative analysis (19–23). Zinner et al. had two eligible groups from the same study, meaning that 6 groups were present in the analysis (23).

A total of 58 participants were included (22 females, age = 26.7 ± 4.0), all were either sedentary or recreationally active, healthy individuals (Table 1) (60). The participants from Rissanen, et al. (21), were a healthy group used as control for a larger study on diabetic patients. Training type included continuous training (*n* = 17), and interval training (*n *= 41). Exercise mode included cycling (*n* = 40), arm and leg cycling exercise (*n *= 10), and unsupervised training (i.e., no reporting of exercise mode) (*n *= 8).

**Table 1:** Summary of participant characteristics by study.

Study	*n*	Females	Age (years)	Body mass (kg)	BMI (kg · m^2^)
Caen et al. (19)	11	–	22 ± 1.2	76 ± 4.0	23.1
Keramidas et al. (20)	10	7	23 ± 4.7	68 ± 18.7	23.7
Kime et al. (68)	9	3	27 ± 5.0	62 ± 12.2	21.9
Rissanen et al. (21)	8	0	38 ± 7.1	86 ± 13.0	26.3
Zinner et al. (23) (Arm + Leg)	10	6	24 ± 2.0	68 ± 9.1	22.0
Zinner et al. (23) (Leg)	10	6	–	69 ± 10.3	–

Data displayed as mean ± standard deviation.

The completed PEDro scores for the included studies can be found in Supplementary 1. No form of blinding was used in any of the included studies. All studies included were sufficiently similar at baseline and used measures of point estimates and variability for the primary outcome measure. The included participant data from Rissanen et al., was a control healthy group from a larger clinical study, and five participants were excluded *post hoc* from the control to match the clinical group in anthropometric and V˙O_2_peak data (21). The data were excluded both at baseline and post-training and did not affect the comparison. Therefore, there was not sufficient follow-up for those initially allocated to the training intervention.

**Figure 2:**
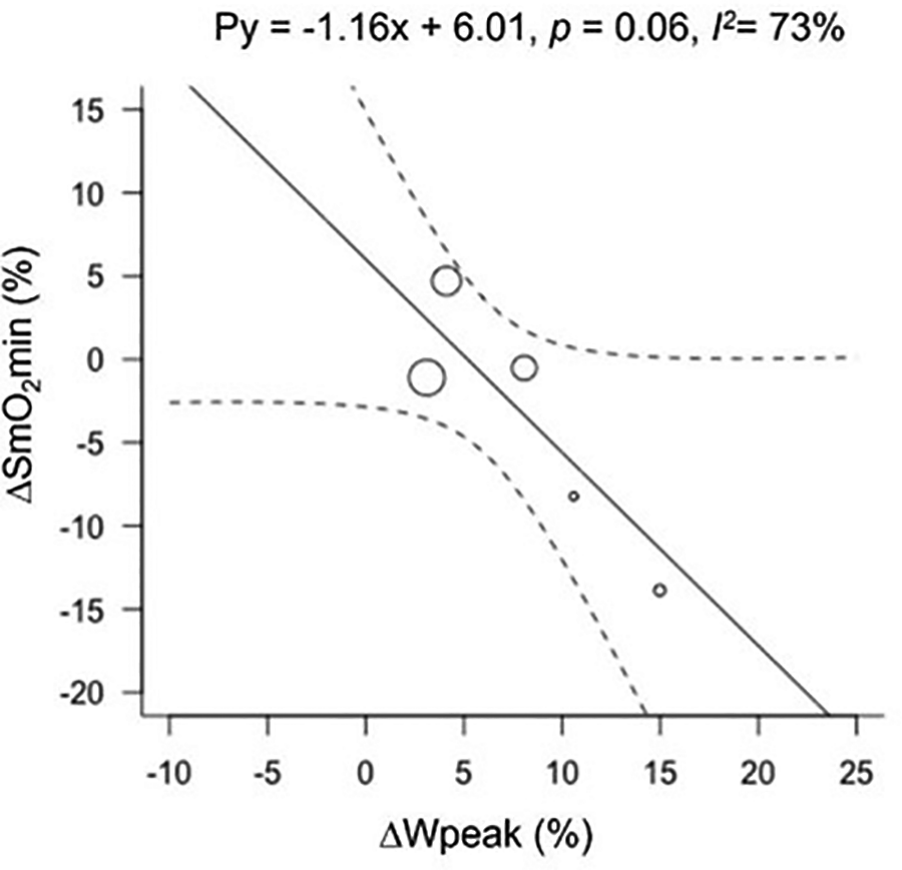
Bubble plot for ΔSmO_2_min with ΔWpeak as a modifier. Change in muscle oxygen saturation minimum (ΔSmO_2_min), and peak power output (ΔWpeak).

For individual studies, the effect of endurance training on SmO_2_min was presented as means and SDs in Table 2, and as effect measures and confidence intervals in Figure 2.

**Table 2:** The effects of endurance training on SmO_2_min pre- and post-training at maximal task tolerance during an incremental exercise test when baseline SmO_2_ is standardized to 100%.

Study	Device	SmO_2_min pre-training	SmO_2_min post-training	ΔSmO_2_min	ΔSmO_2_min SE	*p-*value
Caen et al. (19)	NIRO-200NX	82 ± 5.9	82 ± 3.6	−0.5 ± 9.3	2.8	0.86
Keramidas et al. (20)	InSpectra325	85 ± 4.7	71 ± 5.1	−13.9 ± 19.4	6.1	0.05
Kime et al. (68)	ASTEM Co	80 ± 6.6	74 ± 16.6	−6.1 ± 7.9	2.6	0.05
Rissanen et al. (21)	Oxymon	76 ± 10.8	69 ± 20.7	−8.2 ± 21.3	7.5	0.31
Zinner et al. (23) (Arm + Leg)	NIRO-200NX	83 ± 7.1	88 ± 6.1	4.7 ± 6.6	2.1	–
Zinner et al. (23) (Leg)	NIRO-200NX	87 ± 6.6	85 ± 7.6	−1.1 ± 1.5	0.5	–

Data displayed as mean ± standard deviation. Muscle oxygen saturation (SmO_2_), minimum muscle oxygen saturation (SmO_2_min), difference between SmO_2_min in IET pre- and post-training (ΔSmO_2_min); and standard error of the mean (SE). *p*-value for Zinner, et al. (23), were not provided by the authors.

*p *< 0.05.

No significant effect of endurance training on ΔSmO_2_min was observed. The pooled effect size was −1.92 (95% CI = −5.56 to 1.72, *p* < 0.3), indicating no effect of training on ΔSmO_2_min. There was a high degree of heterogeneity in the pooled results (*I*^2^ = 70%) for changes in ΔSmO_2_min. To investigate the source of heterogeneity in ΔSmO_2_min, we considered all the variables that were extracted. However, a meta-regression was done on Wpeak and V˙O_2_peak only due to their relevance to our question.

### Meta-regression analyses

**Figure 3:**
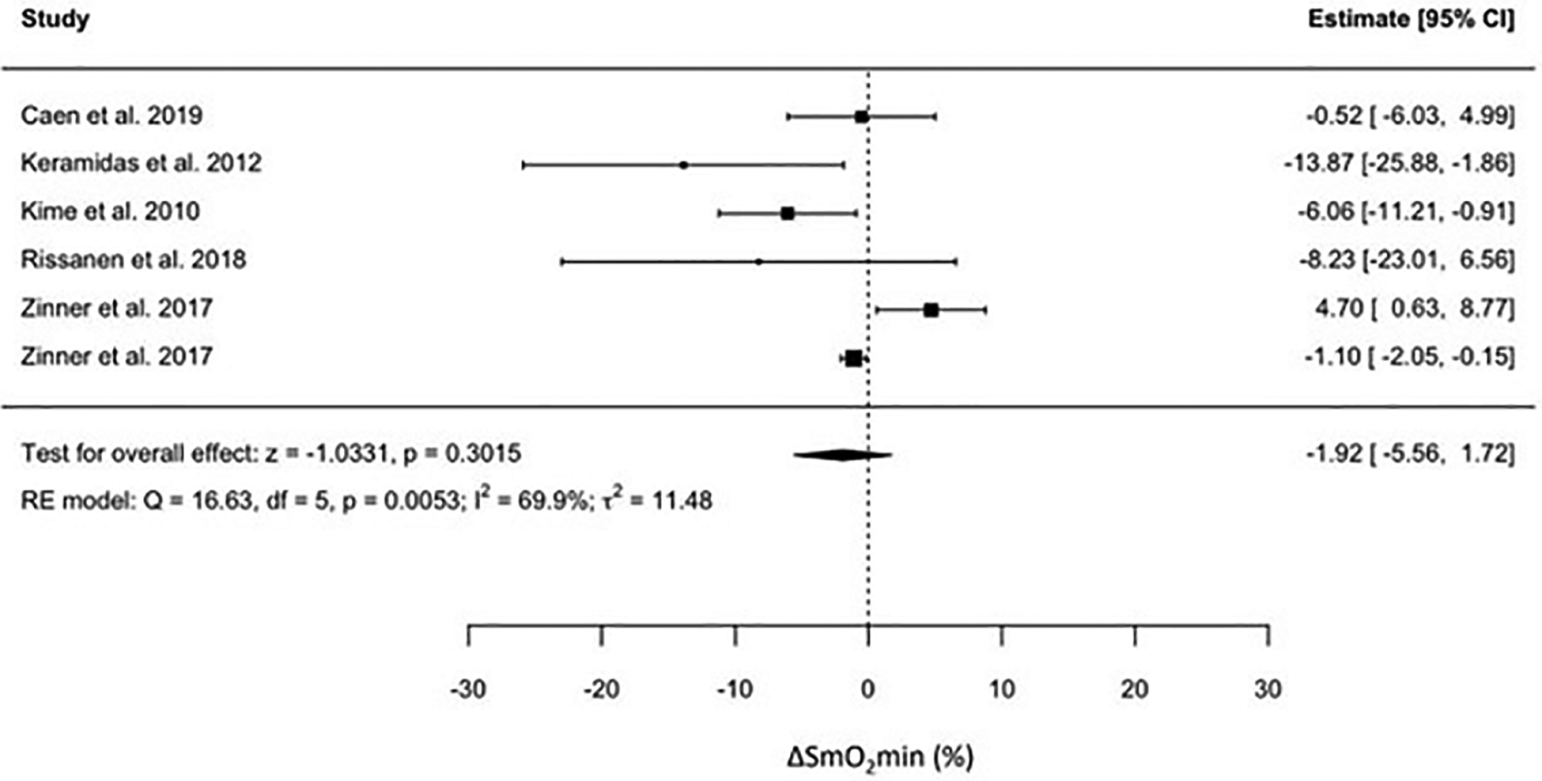
Forest plot of the effect of endurance training on the change in minimum muscle oxygen saturation across the included studies.

For analyses relating to Wpeak, only 4 studies and 5 groups were included; Kime et al. 68) did not report any measures of power. The effect of endurance training on the relationship between Wpeak and ΔSmO_2_min resulted in a tendency for SmO_2_ to be lower with increased Wpeak (Figure 3). The effect of training on Wpeak showed a significant improvement from 286 ± 45 watts (W) to 308 ± 53 W (*p* < 0.01). The heterogeneity for ΔSmO_2_min increased (*I^2^ *= 73%) when grouped with Wpeak. Peak power output data can be found in Supplementary 1.

The relationship between V˙O_2_peak and ΔSmO_2_min showed no effect of training. Keramidas et al. (20) did not state a *p*-value for V˙O_2_peak for the difference between pre-training to post-training. As such, we were unable to calculate the SD of the difference in V˙O_2_peak between the two time points, and it was excluded from the meta-regression. The heterogeneity for ΔSmO_2_min slightly decreased but remained moderate (*I^2^* = 68%) when grouped with V˙O_2_peak. V˙O_2_peak (ml · kg^−1^ · min^−1^) values from individual studies can be found in the Supplementary 1.

### Stratified analyses

Our analysis detected no effect of training on ΔSmO_2_min with regards to sex reported for participants (female or male), training type (continuous or interval), and exercise mode (cycling, arm and leg cycling, or unsupervised training). The heterogeneity for all stratified analyses increased from moderate to high, suggesting that these characteristics would likely not influence the change in ΔSmO_2_min following a training program. For sex, training type, and exercise mode, the *I^2^* increased to 93%, 91%, and 90%, respectively. A summary table of the results, including the *p*-value for interactions for each of the modifiers, can be found in Supplementary 1.

### Between-study bias assessment

**Figure 4:**
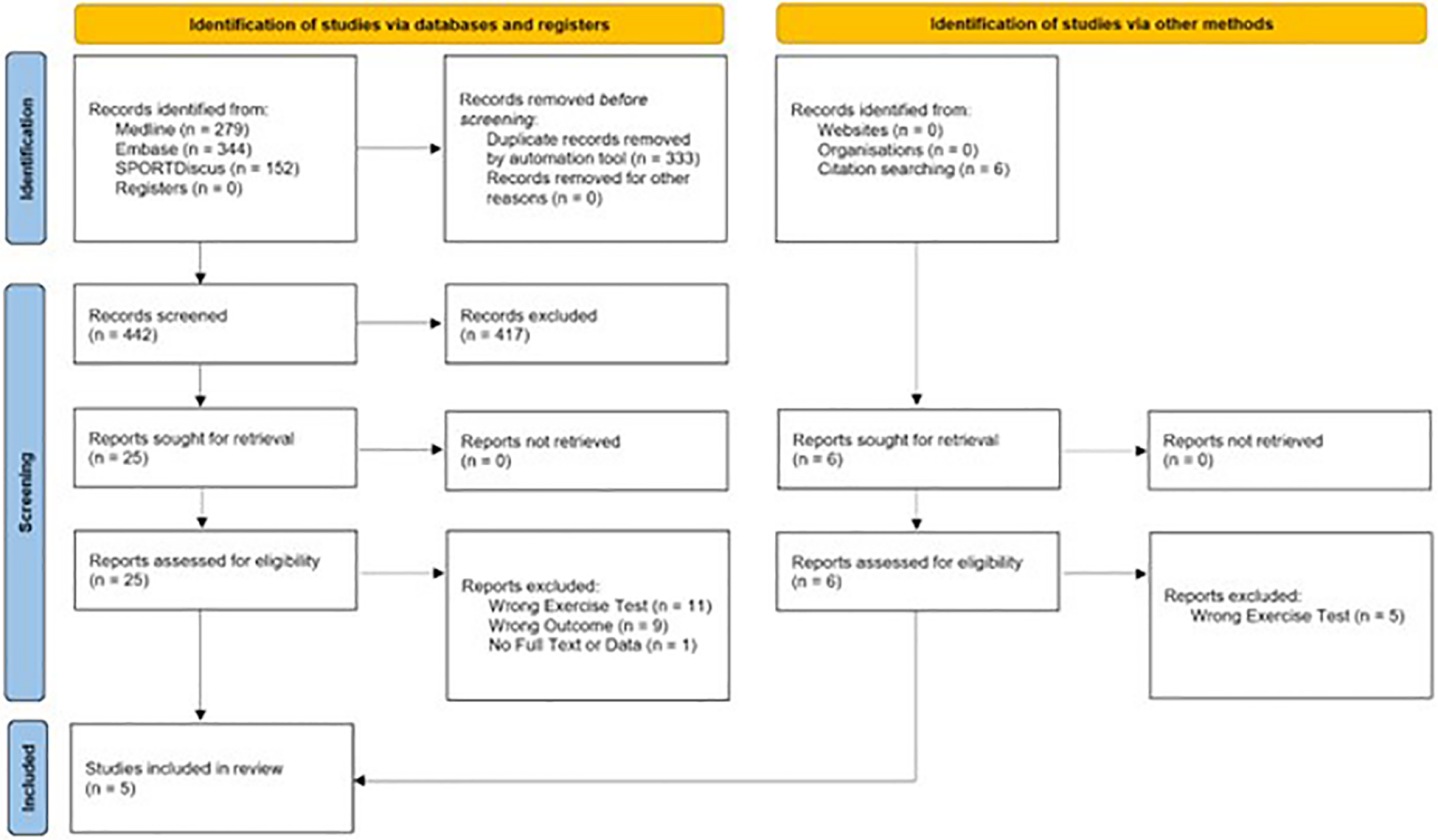
PRISMA 2020 flow diagram.

Visual inspection of a funnel plot (Figure 4) observing changes in ΔSmO_2_min yielded no signs of asymmetry. Additionally, an Egger's test was used to confirm the lack of small sample size bias and test for funnel plot asymmetry (*z *= −1.55, CI = −5.07 to 9.74, and *p *= 0.12) (36).

## Discussion

The aim of our systematic review and meta-analysis was to investigate the effect of endurance training on SmO_2_min and its relationship with any changes in Wpeak and V˙O_2_peak. From our results, we rejected our hypothesis that a greater SmO_2_ desaturation to a lower SmO_2_min, would occur during an IET following endurance training. Three studies demonstrated either a high degree of variance in their results (20, 21), or a higher absolute SmO_2_min, and a reduced ΔSmO_2_min during an IET following training (23). Rissanen et al. (21), included a year-long, unsupervised training intervention, resulting in greater variance in SmO_2_min relative to studies with a controlled training program. Zinner et al., was the only study that included a group with both arm and leg cycling training and was the only study demonstrating an increase in SmO_2_min (i.e., less desaturation) measured at the quadricep at the end of an IET following training (23). Omitting this subgroup from the analysis did not change the results for ΔSmO_2_min. Lastly, Keramidas et al., showed a large CI; however, performance as measured by V˙O_2_peak did not significantly change from pre-training (38.0 ± 5.5 ml · kg^−1^ · min^−1^) to post-training (36.4 ± 4.3 ml · kg^−1^ · min^−1^) (20). This finding contrasts with their Wpeak results that significantly increased from 207 ± 61 to 238 ± 77 W. This is the only study in which Wpeak and V˙O_2_peak did not respond similarly. To further explore the interaction between changes in ΔSmO_2_min and performance measures such as Wpeak and V˙O_2_ separately, subgroup analyses were conducted.

### Subgroup analyses

Heterogeneity increased across all stratified subgroup analyses, with no effect of training. With regards to the effect of sex, our data included 22 female participants out of 58, in four of the six included groups (20, 23, 61). Previous studies looking at sex-based differences indicated that higher adiposity at the quadricep muscles seen more commonly in female participants, was an important factor affecting muscle oxygenation outcomes (15, 62, 63). None of the included studies reported adiposity for males and females independently, which may explain the increased heterogeneity when stratifying the studies by sex.

The exercise type was divided into continuous or interval training. This analysis showed no effect of training on ΔSmO_2_min, with a greater heterogeneity relative to the primary analysis. The continuous training intervention was used in two studies (21, 61). Rissanen et al., performed a year-long training intervention that included both endurance and resistance training. The exercise modalities used in the study were not specified. Therefore, we cannot conclude whether interval training was used, or to what degree the intervention differed from other studies that used interval training as their primary mode of training.

For exercise mode, no effect of training was found. The dominant mode of exercise in the included studies was cycling, both in training and during the IET. Zinner, et al., was the only study that included both leg and arm cycling (23). They included two groups that were exposed to a similar training load, one with leg and arm cycling, and the other with leg only. Interestingly, despite a lack of significant change in their SmO_2_min response after training, their results for the leg and arm group were the only ones showing directionally different findings (increase in SmO_2_min post-training) in the vastus lateralis muscle oxygenation response during an IET. Their report raises an interesting consideration in light of the findings presented in this study, concerning what NIRS can reveal about local skeletal muscle adaptations across muscle groups, or between upper and lower body muscles, during whole-body exercise. The ability of NIRS to quantify SmO_2_ across multiple muscle sites is currently underexplored and may shed light on the integrated systemic and local responses to training interventions. The ability to critically evaluate the effect of exercise mode on SmO_2_ is currently limited due to most studies using cycling as their primary mode of IET, even if the training intervention included a different mode of exercise.

Despite not finding a significant effect of training on ΔSmO_2_min associated with an increase in Wpeak in an IET after training, a trend was detected showing that with improvements in Wpeak, ΔSmO_2_min tended to be greater (i.e., greater desaturation to a lower SmO_2_min post-training). This finding corresponds with previous studies that investigated training interventions and their effects on muscle oxygenation measured by non-IET protocols (43, 56, 64). The most recent study by Paquette et al., investigated the effect of a 3-week training camp on national-level kayakers using muscle oxygen saturation as a primary outcome. Their results showed a greater ability to desaturate to a lower SmO_2_min after training, implying enhanced muscle oxygen extraction in conjunction with an increase in performance (14). The increase observed in Wpeak in the current meta-analysis shows that IET performance was improved with training, with longer duration to maximum exercise tolerance. Thus, despite only observing a tendency for decreased SmO_2_min at the end of the IET (*p *= 0.06), the rate of SmO_2_ desaturation during the IET was slowed after endurance training, producing the same or lower minimum at a higher Wpeak.

It is important to note that despite showing a trend for a greater SmO_2_ desaturation at a higher Wpeak, V˙O_2_peak did not present the same trend. This meta-analysis did not attempt to evaluate how SmO_2_ changes might be related to either peripheral skeletal muscle or systemic cardiopulmonary adaptations. Our results do raise an important question about implications concerning SmO_2_ changes in relation to improvements in performance without evidence of an effect of endurance training on the relationship between peripheral muscle measurements and systemic measures of maximal aerobic capacity.

From the meta-regression of ΔSmO_2_min and V˙O_2_peak, our results show no relationship between the change in ΔSmO_2_min to the change in V˙O_2_peak from pre- to post-training. As mentioned previously, SmO_2_min is associated with maximal task tolerance during an IET (11, 12). This is due to the progressive disruption of local metabolic milieu with increasing workload (2). Our results suggest that a similar SmO_2_min value will be achieved at maximal exercise tolerance after a training intervention, regardless of improvements in fitness. This suggests that a higher SmO_2_ value observed at a similar absolute submaximal workload after a training intervention may be associated with enhanced fitness and predictive of increased performance. For athletes and performance specialists, instead of requiring maximal testing to confirm fitness changes, SmO_2_ could be evaluated more frequently at submaximal workloads to suggest directional performance changes. This is like the current use of heart rate monitors in-field training relative to objective, external load measures such as running pace or cycling power output. Ideally, NIRS could be used in conjunction with heart rate monitors during regular training bouts instead of dedicated testing sessions, to improve tactical decision-making by practitioners and athletes. In this context, by observing the relationship of SmO_2_ to cycling power output, performance capacity could be estimated on a session-to-session basis. Before doing so, future studies should investigate the test-retest reliability of NIRS signals, especially when choosing wearable NIRS sensors during training sessions, to determine the minimal worthwhile change that could indicate fitness improvements.

### Limitations

Our meta-analysis includes a small number of studies, and limited number of participants. Despite not presenting a risk of bias between studies, a lack of standardization of training intervention may have influenced our results and contributed to the heterogeneity of the analysis (65). In line with this limitation, inherent differences in exercise intensity during the training period may have effected training adaptations. One of the studies defined their intervention as endurance training but did not standardize their training intervention (21). For exercise mode, as indicated previously, cycling is a common exercise mode for IET; however, SmO_2_ responses may be different for running, rowing, and other exercise modalities related to different muscle recruitment demands. Future studies should use the same mode for IET as for training. Additionally, NIRS signals are sensitive to the absorbance characteristics of biological tissues (15, 62, 66, 67). Therefore, for better quantification of training intervention effects on SmO_2_, reporting adiposity is critical. Lastly, the focus of the analysis was to quantify the effects of training on SmO_2_min during an IET; however, future studies should consider investigating the ability of other testing protocols to quantify muscle oxygenation, such as constant workload protocols across intensity domains and intermittent IETs, among other protocols.

## Conclusion

From our results, we concluded that endurance training does not affect the SmO_2_min value attained at the end of an IET. These findings are especially interesting when observing the effect of endurance training on the relationship between ΔSmO_2_min, Wpeak, and V˙O_2_peak. A trend found for an effect of training on the relationship between ΔSmO_2_min to Wpeak may suggest that any changes in SmO_2_ are best seen during submaximal exercise, rather than at maximal task tolerance. Interestingly, no effect of training on the relationship between ΔSmO_2_min and V˙O_2_peak was observed. This finding raises an important question about whether SmO_2_min is indicative of systemic cardiovascular changes, or peripheral muscle adaptations to endurance training. For practical applications, our findings suggest that NIRS provides a useful tool to quantify muscle adaptations relative to increased power output and, despite not showing any significant changes following endurance training, it may still be used as an additional physiological marker of exercise tolerance during an IET.

## Data Availability

The raw data supporting the conclusions of this article will be made available by the authors, without undue reservation.
