# Supplementary material for: The effects of endurance training on muscle oxygen desaturation during incremental exercise tests: a systematic review and meta-analysis
Source: Front Sports Act Living. 2024 Oct 24;6:1406987. doi: 10.3389/fspor.2024.1406987 (PMC11540711; doi:10.3389/fspor.2024.1406987)
Supplement: Supplementary file 1 [file Datasheet1.zip › NIRS Results.docx]

**Forest plot**


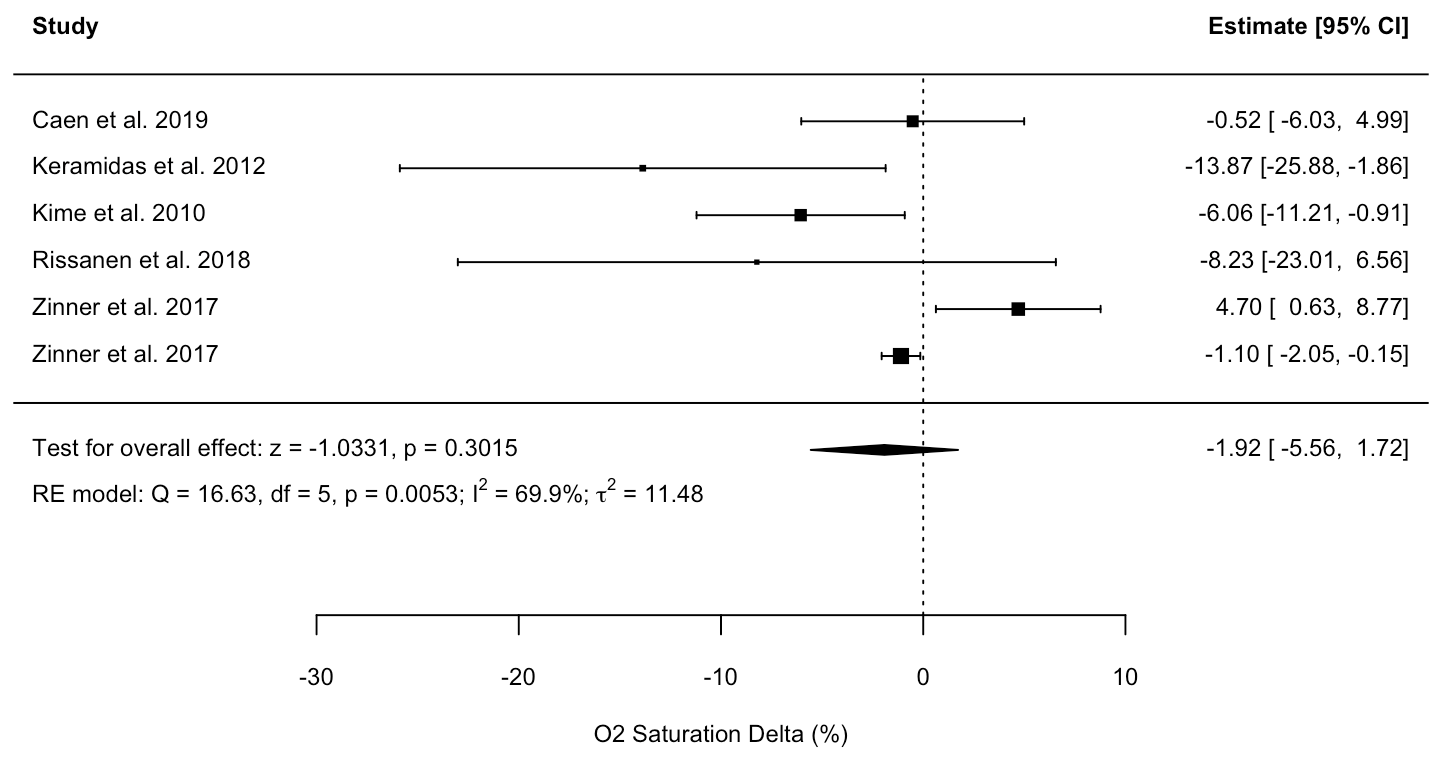


**Funnel Plot**
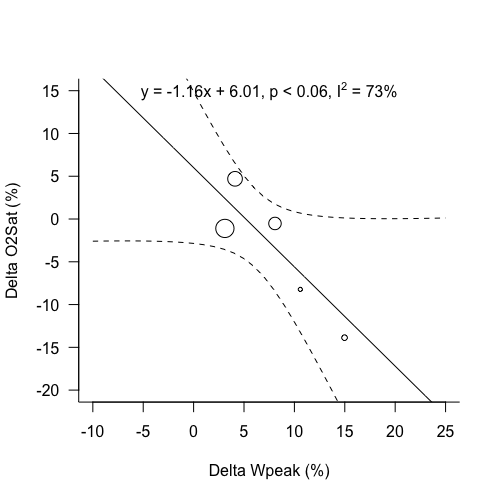


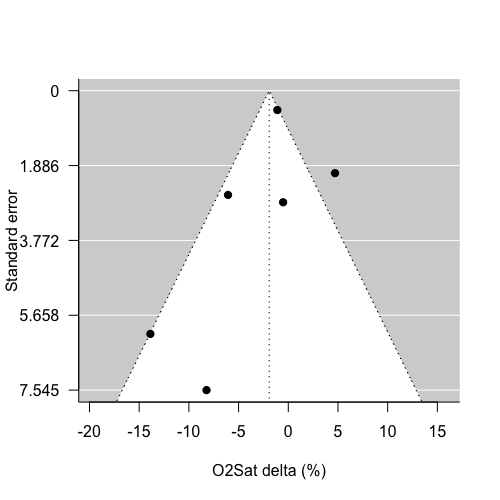


**Egger’s Test**

Test for Funnel Plot Asymmetry: z = -1.5477, p = 0.1217

Limit Estimate (as sei -> 0): b = 2.3345 (CI: -5.0689, 9.7379)

**Bubble plot with Wpeak delta modifier**

**Bubble plot with VO2peak delta modifier**


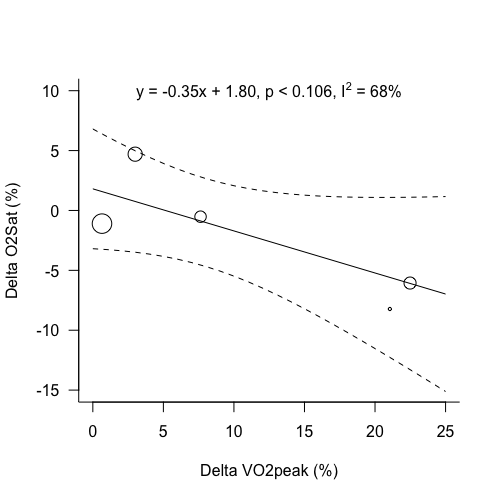


**Sex as a modifier**

> MetaRegressionSubgroupEstimates

Mixed-Effects Model (k = 6; tau^2 estimator: DL)

tau^2 (estimated amount of residual heterogeneity): 30.1793 (SE = 27.4193)

tau (square root of estimated tau^2 value): 5.4936

I^2 (residual heterogeneity / unaccounted variability): 93.04%

H^2 (unaccounted variability / sampling variability): 14.36

Test for Residual Heterogeneity:

QE(df = 4) = 57.4516, p-val < .0001

Test of Moderators (coefficients 1:2):

QM(df = 2) = 2.6788, p-val = 0.2620

Model Results:

estimate se zval pval ci.lb ci.ub ​

factor(Dataset$SexDescription)1.Both -4.1156 4.1952 -0.9810 0.3266 -12.3380 4.1068

factor(Dataset$SexDescription)3.Male -3.7565 2.8673 -1.3101 0.1902 -9.3762 1.8633

---

Signif. codes: 0 ‘***’ 0.001 ‘**’ 0.01 ‘*’ 0.05 ‘.’ 0.1 ‘ ’ 1

> MetaRegressionSubgroupDifference

Mixed-Effects Model (k = 6; tau^2 estimator: DL)

tau^2 (estimated amount of residual heterogeneity): 30.1793 (SE = 27.4193)

tau (square root of estimated tau^2 value): 5.4936

I^2 (residual heterogeneity / unaccounted variability): 93.04%

H^2 (unaccounted variability / sampling variability): 14.36

R^2 (amount of heterogeneity accounted for): 0.00%

Test for Residual Heterogeneity:

QE(df = 4) = 57.4516, p-val < .0001

Test of Moderators (coefficient 2):

QM(df = 1) = 0.0050, p-val = 0.9437

Model Results:

estimate se zval pval ci.lb ci.ub ​

intrcpt -4.1156 4.1952 -0.9810 0.3266 -12.3380 4.1068

factor(Dataset$SexDescription)3.Male 0.3591 5.0814 0.0707 0.9437 -9.6003 10.3185

**Exercise Type as a modifier**

> MetaRegressionSubgroupEstimates

Mixed-Effects Model (k = 6; tau^2 estimator: DL)

tau^2 (estimated amount of residual heterogeneity): 22.4332 (SE = 20.7307)

tau (square root of estimated tau^2 value): 4.7364

I^2 (residual heterogeneity / unaccounted variability): 90.72%

H^2 (unaccounted variability / sampling variability): 10.78

Test for Residual Heterogeneity:

QE(df = 4) = 43.1080, p-val < .0001

Test of Moderators (coefficients 1:2):

QM(df = 2) = 4.4642, p-val = 0.1073

Model Results:

estimate se zval pval ci.lb ci.ub ​

factor(Dataset$TypeDescription)1.Interval -2.2788 2.5074 -0.9088 0.3634 -7.1933 2.6357

factor(Dataset$TypeDescription)2.Continous -7.0470 3.6945 -1.9074 0.0565 -14.2882 0.1941 .

---

Signif. codes: 0 ‘***’ 0.001 ‘**’ 0.01 ‘*’ 0.05 ‘.’ 0.1 ‘ ’ 1

> MetaRegressionSubgroupDifference

Mixed-Effects Model (k = 6; tau^2 estimator: DL)

tau^2 (estimated amount of residual heterogeneity): 22.4332 (SE = 20.7307)

tau (square root of estimated tau^2 value): 4.7364

I^2 (residual heterogeneity / unaccounted variability): 90.72%

H^2 (unaccounted variability / sampling variability): 10.78

R^2 (amount of heterogeneity accounted for): 5.54%

Test for Residual Heterogeneity:

QE(df = 4) = 43.1080, p-val < .0001

Test of Moderators (coefficient 2):

QM(df = 1) = 1.1404, p-val = 0.2856

Model Results:

estimate se zval pval ci.lb ci.ub ​

intrcpt -2.2788 2.5074 -0.9088 0.3634 -7.1933 2.6357

factor(Dataset$TypeDescription)2.Continous -4.7682 4.4651 -1.0679 0.2856 -13.5196 3.9832

**Exercise Mode as a modifier**

> MetaRegressionSubgroupEstimates

Mixed-Effects Model (k = 6; tau^2 estimator: DL)

tau^2 (estimated amount of residual heterogeneity): 19.8682 (SE = 20.3893)

tau (square root of estimated tau^2 value): 4.4574

I^2 (residual heterogeneity / unaccounted variability): 90.41%

H^2 (unaccounted variability / sampling variability): 10.43

Test for Residual Heterogeneity:

QE(df = 3) = 31.2940, p-val < .0001

Test of Moderators (coefficients 1:3):

QM(df = 3) = 7.8713, p-val = 0.0487

Model Results:

estimate se zval pval ci.lb ci.ub ​

factor(Dataset$ExerciseModeDescription)0.Unknown -8.2270 5.2357 -1.5713 0.1161 -18.4889 2.0348

factor(Dataset$ExerciseModeDescription)1.Cycling -4.9955 2.3827 -2.0966 0.0360 -9.6656 -0.3255 *

factor(Dataset$ExerciseModeDescription)2.FullBody 4.7000 4.6846 1.0033 0.3157 -4.4817 13.8817

---

Signif. codes: 0 ‘***’ 0.001 ‘**’ 0.01 ‘*’ 0.05 ‘.’ 0.1 ‘ ’ 1

> MetaRegressionSubgroupDifference

Mixed-Effects Model (k = 6; tau^2 estimator: DL)

tau^2 (estimated amount of residual heterogeneity): 19.8682 (SE = 20.3893)

tau (square root of estimated tau^2 value): 4.4574

I^2 (residual heterogeneity / unaccounted variability): 90.41%

H^2 (unaccounted variability / sampling variability): 10.43

R^2 (amount of heterogeneity accounted for): 16.34%

Test for Residual Heterogeneity:

QE(df = 3) = 31.2940, p-val < .0001

Test of Moderators (coefficients 2:3):

QM(df = 2) = 4.2580, p-val = 0.1190

Model Results:

estimate se zval pval ci.lb ci.ub ​

intrcpt -8.2270 5.2357 -1.5713 0.1161 -18.4889 2.0348

factor(Dataset$ExerciseModeDescription)1.Cycling 3.2315 5.7524 0.5618 0.5743 -8.0430 14.5060

factor(Dataset$ExerciseModeDescription)2.FullBody 12.9270 7.0256 1.8400 0.0658 -0.8428 26.6969

*

Measure, estimate, p-value (at end), confidence into table, modifier
